# Mental health support across the sight loss pathway: a qualitative exploration of eye care patients, optometrists, and ECLOs

**DOI:** 10.1038/s41433-022-02373-z

**Published:** 2023-01-10

**Authors:** M. Trott, R. Driscoll, R. Bourne, J. Slade, H. Ingleton, S. Farrell, M. Bowen, R. Lovell-Patel, J. Kidd, S. Pardhan

**Affiliations:** 1 Vision and Eye Research Institute, Anglia Ruskin University, Cambridge, UK; 2 Centre for Public Health, Queen’s University Belfast, Belfast, UK; 3 Royal National Institute of Blind People, London, UK; 4 Cambridge University Hospitals NHS Foundation Trust, Addenbrooke’s Hospital, Cambridge, UK; 5 College of Optometrists, London, UK; 6 NHS England and NHS Improvement, London, UK

**Keywords:** Health occupations, Rehabilitation

## Abstract

**Background:**

The process of becoming visually impaired or blind is undoubtedly a highly emotional experience, requiring practical and psychological support. Information on mental health support provision in the UK across the sight-loss pathway, however, is largely unknown, especially amongst healthcare practitioners that are often sought after for advice: the referring optometrist and eye clinic liaison officer (ECLO). This study aims to ascertain the perceived accessibility and quality of mental health support across the sight-loss pathway.

**Methods:**

Semi-structured individual interviews were conducted with patients with a diagnosed eye condition who had received care from a hospital eye service, referring optometrists, and ECLOs. Following interview transcription, results were synthesised in a narrative analysis.

**Results:**

A total of 28 participants were included in the analysis, of which 17 were participants with various eye conditions, five were referring optometrists, and five were ECLOs. After analysis, three broad themes emerged: (1) The emotional trauma of diagnosis (2) Availability of mental health support; (3) The point where mental health support is most needed across the sight-loss pathway. Several patients reporting that they had received no offer of support nor were they signposted to any possible sources. Referring optometrists and ECLO’s agreed.

**Conclusion:**

It is important that referring optometrists are aware of the need for mental health support services and can signpost to local support services including the third sector anytime during the referral process. Future large-scale, UK-wide research into referral practice and signposting for mental health support for patients is warranted, to identify how services can be improved in order to ensure that the wellbeing of patients is maintained.

## Introduction

Conditions that affect the eye can yield a wide range of visual outcomes, from normal vision to complete blindness, with varying degrees of progression. Visual impairment has been reported to affect at least 1.1. billion people globally [1, 2], with blindness affecting 36–43 million people, with a further 217–295 million people with moderate to severe visual impairment. The presence of an eye condition, becoming sight impaired, or severely sight impaired is undoubtedly a highly emotional experience [3]. For example, visual impairment has been independently associated with several mental health conditions, including depression [4–7], anxiety [5, 6, 8], and post-traumatic stress disorder [9]. Several studies report that eye care patients experience degrees of emotional trauma and may require some form of mental health support [10, 11] which should not be limited to patients with recent diagnoses of an eye condition [10, 12, 13]. In addition, it has been shown that anxiety is one of the main reasons for clinic non-attendance at eye clinics [14–17].

It has been recommended that support should be provided early in the eye care pathway, as patients often do not seek mental health assistance until they have reached a crisis point [10]. Although mental health support (including emotional support, counselling, patient support groups, primary care, psychology services, secondary care psychiatry services), has been shown to be beneficial to the wellbeing of patients with visual impairment [18], the current provision of mental health support to patients across the eye care pathway in the UK is largely unknown. There is some evidence to suggest that Eye Clinic Liaison Officers (ECLOs) do provide valuable mental health support [19, 20], however not all eye clinics across the UK have these roles, so these services are not universal. In addition, it is not known how much mental health signposting referring practitioners (such as optometrists) offer, or indeed what the perceived accessibility and quality of mental health support across the eye care pathway is from the perspective of the patient or hospital eye care staff, or what priority people with an eye related diagnosis are given within the triage systems for mental health care. The aim of this preliminary study, therefore, is to examine the experiences of mental health provision across the eye care pathway, from several different perspectives, including eye care patients, referring optometrists, and ECLOs. This group of healthcare professionals is particularly important because referring professionals have the opportunity to refer patients to mental health services at an early stage in the eye care pathway, and ECLOs are frequently asked about the provision of support as part of their role. This has the potential to inform future research and identifying relevant policy changes.

## Materials/subjects and methods

Semi-structured interviews were conducted with people with various eye conditions (including patients registered as sight impaired, severely sight impaired, and patients with no sight loss registration), ECLOs, and referring optometrists.

Participants were recruited purposively via external advertisements through the third sector, participants from previous eye related research (who had previously consented to be contacted for future research), word of mouth, and marketing leaflets. The study was approved by the Anglia Ruskin University School of Medicine Ethics Panel (MED-SREP-21-003). All participants provided informed consent, including explicit consent for quotes to be used in academic publications.

Each interview lasted for approximately 30 minutes and was conducted by the same researcher (MT) to minimise inter-rater variability. Participants were asked open-ended questions about their experiences of care across all points of their eye care pathway (see Supplementary materials for interview questions), access to mental health support (this was intentionally worded as broadly as possible to capture the widest array of answers possible) across the pathway, and where they felt improvements could be made regarding mental health support. Specifically, each patient was asked to describe their experiences of eye care, from initial symptoms, initial referral, diagnosis, hospital treatment (if relevant), and post-acute care. Practitioners were asked to describe the eye care pathway and were then asked to describe their experiences of each stage, with a particular focus on areas to be improved.

Each interview was transcribed and independently checked by another (RD, MT) researcher. Following transcription, an analysis of answers was conducted independently by two researchers (MT, RD), using NVivo (Version 12) software. Following analysis, the results were synthesised in a narrative analysis.

## Results

A total of 28 participants were included in the analysis, of which 18 were patients with varying eye conditions, 5 were referring optometrists, and 5 were ECLOs.

After analysis, three broad themes emerged:

1. The emotional trauma of diagnosis for the patient and family.
2. Lack of signposting for mental health support.
3. Which point mental health support is most needed across the sight-loss pathway.

### Emotional trauma of diagnosis for the patient and family

Several patients commented on the emotional trauma of the point of diagnosis, and the need for mental health support:

*‘We do feel a bit abandoned, I’m sure everybody does*‘ – *PAT1 (AMD)*

‘It gets to me sometimes. Sometimes it really gets to me’ – PAT2 (Retinal dystrophy, retinopathy, glaucoma)

This trauma was not limited to the patients—the trauma for families was also mentioned in the context of being diagnosed with a genetic eye condition:

*‘…my family and [I] had an appointment with the genetics team at the hospital…my family were more grieving at the process of finding out that there’s this genetic condition in our family. I don’t know how ready they were to take that information in…some quite emotive irrational decisions about whether to receive the support.’ – PAT3 – (Leber Hereditary Optic Neuropathy (LHON))*

*‘I think it’s a lot to take in and I’m not sure my family were ready to take it in necessarily’ – PAT3 – (LHON)*

### Lack of signposting for emotional support

#### Patients

Although some patients knew where to seek mental health support, others stated that they were not made aware of the potential sources:

*‘Never. I have never been offered any [mental health] support at all’ – PAT8 (glaucoma)*

*‘No [mental health support], nothing if we’re talking eyes, certainly no.’ – PAT10 (detached retina, glaucoma)*

*‘[The eye hospital] also has a counselling service, but nobody tells you about it.’ – PAT4 (Stargardt)*

*‘I know the [eye sight charity] will offer it, but it’s an on-demand service, you have to ask for it, I don’t know that it comes to you automatically’ – PAT5 (AMD)*

*‘When they told me after my sessions that I could continue my [previously received sight loss counselling] sessions privately I was like ‘why – why hasn’t anybody told me this’. I know that this…is not available [as I was going private] to everyone, but it’s a lot better than feeling suicidal.’ – PAT4 (Stargardt)*

Positive patient experience when support was available was noted:

*‘I spoke for a good hour, and I think she was one of the reasons why I didn’t give way to despair… thank God for ECLOs and various charities – I have had counselling from them, friendship from them, and a listening ear.” – PAT6 (glaucoma)*

*‘[attending a support group] was like an emotional support I’d never received before… they were sharing jokes…I think speaking to other blind people, partially sighted people…you get your support by sharing information and you’re not aware that you’re actually supporting each other.’ – PAT7 (glaucoma, retinitis pigmentosa, cataracts)*

#### ECLOs

The poor availability of mental health support was also highlighted by ECLOs, concurring with the patient experience:

*‘I think most people who work in this field would agree there’s not enough provision overall for sight specific counselling.’ ECLO1*

*‘I’m very aware of the real need for more support emotionally.’ – ECLO2*

*‘I don’t think there is enough support.’ – ECLO3*

Some ECLOs reported difficulties in offering support. For example, some ECLOs who were not embedded into the hospital eye service (HES), and stated private space to talk to patients was not always available which limited the level of support they could give, whereas other ECLOs who were embedded in their respective HES could offer more support in a dedicated private room.

#### Referring optometrists

When asked about the provision of mental health support in eye care services, referring optometrists also agreed that emotional support was not always signposted:

*‘It’s dire, utterly dire. There is such limited support’ – OPTOM1*

*‘The emotional side of things is kind of left at the wayside. So I’d say it’s quite poor.’ OPTOM2*

### The point at which mental support is needed in the sight loss pathway

#### Patients

When asked where in the sight loss pathway mental health support should sit, some participants with vision loss stated that they thought it should predominantly sit at the point of diagnosis:

*‘that’s when you need real [mental health] support, those early days when you first find out.’ – PAT1 (AMD)*

#### ECLOs

ECLOs reported the need for mental health support at several stages of the pathway and thought it the responsibility of everybody within the HES to consider support:

*‘Definitely the point of diagnosis is massively important in some cases…but I think it needs to always be available throughout the stages of someone’s journey because people go through that grief and loss cycle in their own time…many people I’ve worked with for many years, who are well established with their vision impairment, well established in the community of vision impairment…may still have a dip at any point in their life where they go back, they coped fantastically and get on with their life but then they have a dip, for many different reasons where they still need emotional support’ – ECLO1*

*‘It’s a bit of responsibility for everybody…dealing with people with vision impairment. You know, from the medics themselves, and I know they don’t have an awful lot of time. It’s a bit like a conveyor belt system. But one thing they could remember to do is perhaps you use a little bit less of the medical terminology in language, [be]cause often, people come away from a session with the doctor and they haven’t really got much of a clue about what was said, you know, or what their condition is, or what things mean for them, or how their treatments going to work, or even how to use eye drops’ – ECLO2*

#### Referring optometrists

Referring optometrists had differing views on where the support was needed most*:*

*‘I think secondary care [is] probably the best place to do this to guide them towards these [mental health] support groups’ – OPTOM4*

*‘Emotional support should sit in the NHS more, rather than the third sector, definitely’ – OPTOM1*

*‘I think that [mental health] support should start both in the primary [and] secondary care’*. – *OPTOM4*

Furthermore, one optometrist was unsure as to where mental health support should sit, and highlighted a need for further training, especially as where to signpost the patients to:

*‘I would say…we need more support to help as optometrists. Where to point patients because you can say, ‘go see your GP’, but then there’s going to be a waiting list…[so] there’s no immediate support. And the charities, I don’t know if they give much emotional support, they’re much more practical based.’ – OPTOM2*

## Discussion

This qualitative study explored the perspectives of need and availability of mental health provision across the eye care pathway from the perspectives of low-vision patients, referring optometrists and ECLOs. Three key themes that arose from the study were (a) the emotional trauma of diagnosis (b) the availability of mental health support, and (c) the point at which mental health support is most needed across the sight-loss pathway.

This study highlights the need for support at the point of diagnosis, concurring with previous studies in this area [10, 11]. Evidence of the availability of mental health support throughout the pathway was not that forthcoming, with many patients reporting that they had not pro-actively received any form of support, and the majority of patients stating that they didn’t know where to go for support should they need it. This is concerning as it has the potential to directly affect a patient’s quality of life. ECLOs and referring optometrists agreed that more needed to be done regarding the provision and signposting of mental health support. Interestingly, referring optometrists felt that perhaps it was not up to them to support or signpost patients for mental health support, whist patients and ECLO’s reported the need for it across the whole vision loss pathway.

One referring optometrist did not know what kind of support was available to patients at the time of referral, and another highlighted the need for further training in this area. It has been indicated previously that patients only self-refer to support services when they are ‘at crisis point’, and that mental health support interventions may be beneficial earlier in the sight loss pathway [10]. Further studies are warranted to determine the level of knowledge referring optometrists have regarding availability and signposting of emotional support services. The lack of signposting is also seen at secondary and tertiary care level, with one study reporting that only 17% of patients were referred by ophthalmologists for mental health services, and only 35% of ophthalmologists stating that they did not struggle with the discussion of psychiatric/psychological problems [21], suggesting that further training may also be warranted in this group of secondary healthcare professionals. Further studies could assess the success of interventional studies that signpost potential low-vision patients to sources of mental health support if needed.

Patients reported that support would be most beneficial at the point of diagnosis, agreeing with some current literature [10, 11], however ECLOs, from their experience, reported that mental health support may be needed at any point of the vision loss pathway, even after the patient has been discharged and has lived successfully with their visual impairment for some time. Currently, the National Institute for Health and Care Excellence (NICE) does not explicitly mention mental health support in its guidelines for glaucoma [22] or cataract [23], but is featured in the guidelines for age-related macular degeneration (AMD) [24]. It is unclear why this discrepancy exists. Moreover, the processes of mental health referral remain unclear—future research is warranted to examine healthcare professionals’ knowledge (and utilisation of) of how to refer patients for further mental health support.

The results of this study should be considered within its limitations. It is possible (likely even) that practice may vary across the regions, although care was taken to draw the sample from as wide a range of geographical locations in England as the scope and scale of the project permitted. However, further larger studies will determine the availability and signposting of mental health services, especially at referral point and at diagnosis. It is clear that mapping the availability and nature of mental health support services for people with low vision should be routinely carried out.

The patients who participated in this study were predominantly affected by glaucoma. This represents a selection bias and is a limitation of the study, although glaucoma has been reported as the 2nd highest cause of severe sight impairment registrations in the UK [25]. It is conceivable that patients with other eye diseases would have a different experience of accessing mental health services as their symptomology and presentation to health care professionals is different. Furthermore, this research was limited regarding the breadth of healthcare professionals interviewed: it is reasonable that different professionals (such as ophthalmologists and rehabilitation officers) could provide key further insights. Lastly, the perspective of mental health services was not measured—which could provide information as to the type of referrals that would be deemed appropriate. Future research is warranted to examine this, as is research to examine the provision of mental health support in more granularity.

This report provides preliminary evidence that the provision of mental health support across the sight-loss pathway is likely to be poor across the UK and is not likely to be being delivered as standard practice in most areas. Several patients reported that they had received no offer or been signposted to any sources (including that of the third sector). Data from clinicians also provided evidence that support services were likely to be poorly signposted and not well understood by those needing this information. There was limited evidence of referring optometrists (primary care) determining effectively whether there was a need for mental health support, and some evidence that further training may be required to support optometrists to do this more effectively, and to understand their role in providing information about local services at the point of referral. It is important that an individual’s need for mental health services is ascertained for patients across the whole eye health and sight loss pathway (in all specialisms—cataract, glaucoma, AMD, diabetic retinopathy, refraction and retinal specialisms), and also possibly beyond. This is likely to require shared standards of care and close cooperation between primary care optometrists and GPs, secondary care optometrists, ophthalmologists, ophthalmic nurses, orthoptists and ECLOs, and secondary care psychiatry and tertiary/community mental health and social care professionals and service providers.

### Summary

#### What was known before

- The diagnosis of an eye condition can be a traumatic experience.
- Visual impairment has been independently associated with several mental health conditions.

#### What this study adds

- There appears to be very little mental health support across the eye care pathway Mental health support is sorely needed at several points in the eye care pathway.
- Referring optometrists need to be aware of the need for mental health support, where this support can be accessed, and how to signpost patients for support at this early stage.

## Supplementary information

Supplementary file

## Data Availability

Anonymised data for this study is available from the corresponding author upon reasonable request.
