# Supplementary material for: Mental health support across the sight loss pathway: a qualitative exploration of eye care patients, optometrists, and ECLOs
Source: Eye (Lond). 2023 Jan 10;37(12):2554–8. doi: 10.1038/s41433-022-02373-z (PMC10397192; doi:10.1038/s41433-022-02373-z)
Supplement: Supplementary file 1 — Supplementary file [file 41433_2022_2373_MOESM1_ESM.docx]

## Patients

1. Can you tell us a little bit about yourself, including age, eye condition, brief history of the eye condition?
2. Can you tell us what treatment you have had, including when (and length of time living with your diagnosed condition)?
3. Has anyone ever spoken to you about your eyecare experience (either researchers or health professionals)
4. How did you first find out about your sight condition? Were you offered any support at this stage? What would have helped you at this time?
5. When you were first diagnosed did you feel you understood your eye condition and what it might mean? Were you offered or told about any mental health support at this time?
6. Regarding your treatment/management of your condition, do you feel you have everything you need? (materials, support, etc).
7. Are you registered as SI or SSI? CVI? If yes, can you tell me a little bit about the certification process?
8. Are you currently in receipt of any social care support?
9. You feel you have needed social care support at any point in your eye care journey?
10. I’d now like you to think about the mental health support you received to help you come to terms with your sight condition and adjust to living with sight loss. What support were you offered? By whom? How useful was this? How did you find out about it?
11. Would you have liked any other form of support during this time? Probe for both practical and mental health support.
12. Have you had someone that you can talk to about your experiences about your eye condition? Who was this (friend/family vs professional).
13. Has your eye condition had an impact on your emotional wellbeing or mental health? Have you been referred into any mental health services? How useful was this, or would they have found this useful?
14. In an ideal world how would you find out about the support available to you?
15. What would have helped you the most during your experience of: - (key stages such as initial referral, diagnosis of condition, treatment/management of condition, certification, registration)
16. Where do you feel in your experience the major difficulties have been and how could that have been made easier for you to cope with?
